# Supplementary material for: Decoupling of mRNA and Protein Expression in Aging Brains Reveals the Age-Dependent Adaptation of Specific Gene Subsets
Source: Cells. 2023 Feb 14;12(4):615. doi: 10.3390/cells12040615 (PMC9954025; doi:10.3390/cells12040615)

Supplemental Figure S1

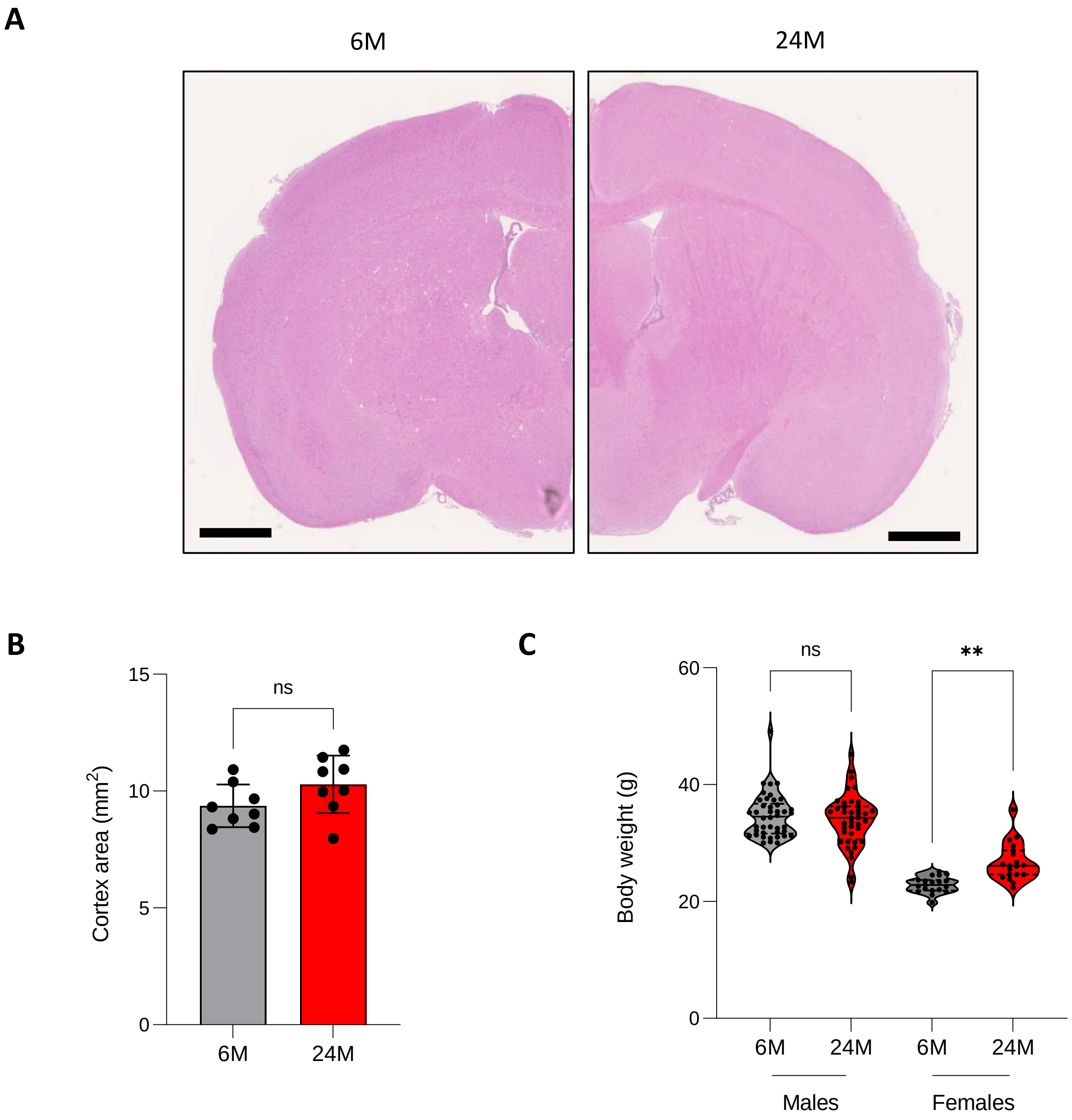

Supplemental Figure S2

A

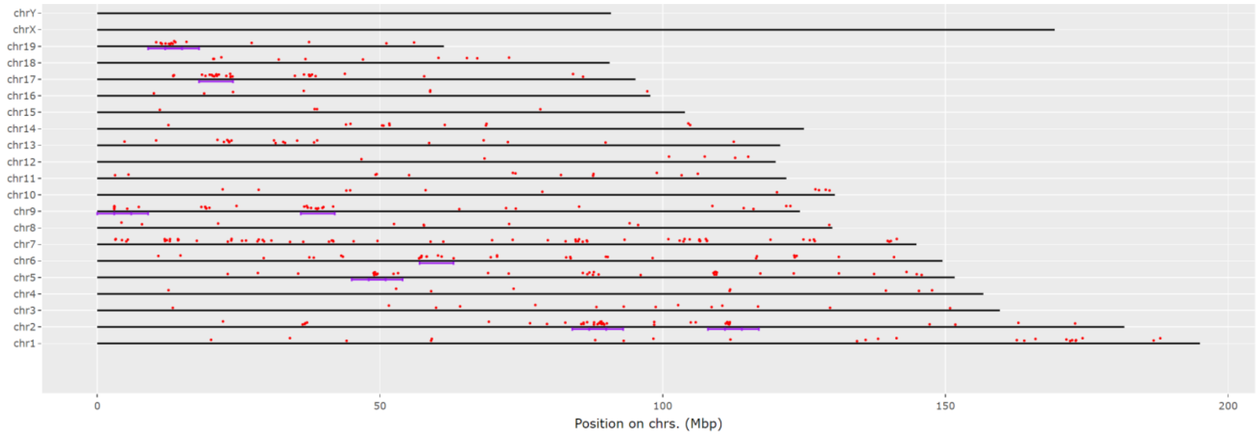

B

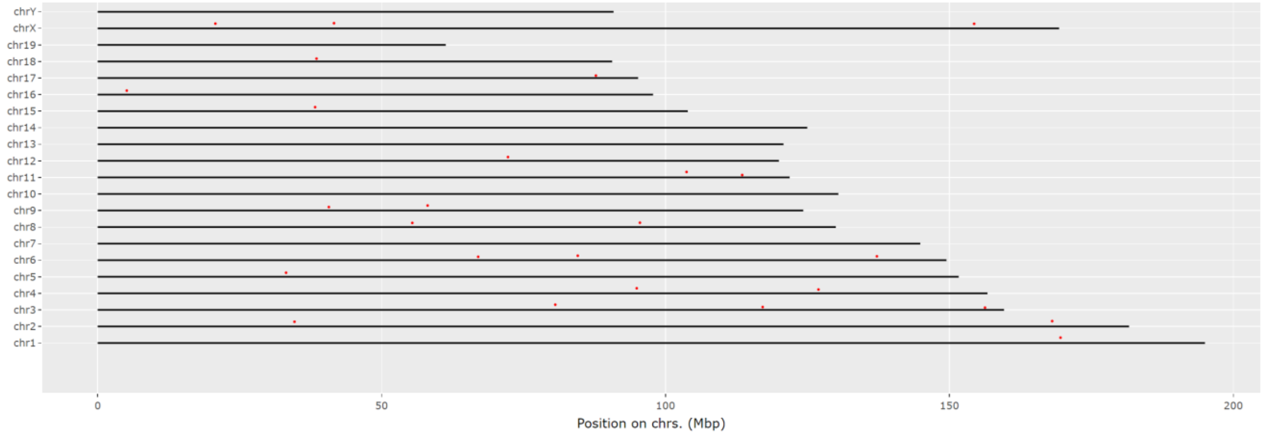

Supplemental Figure S3

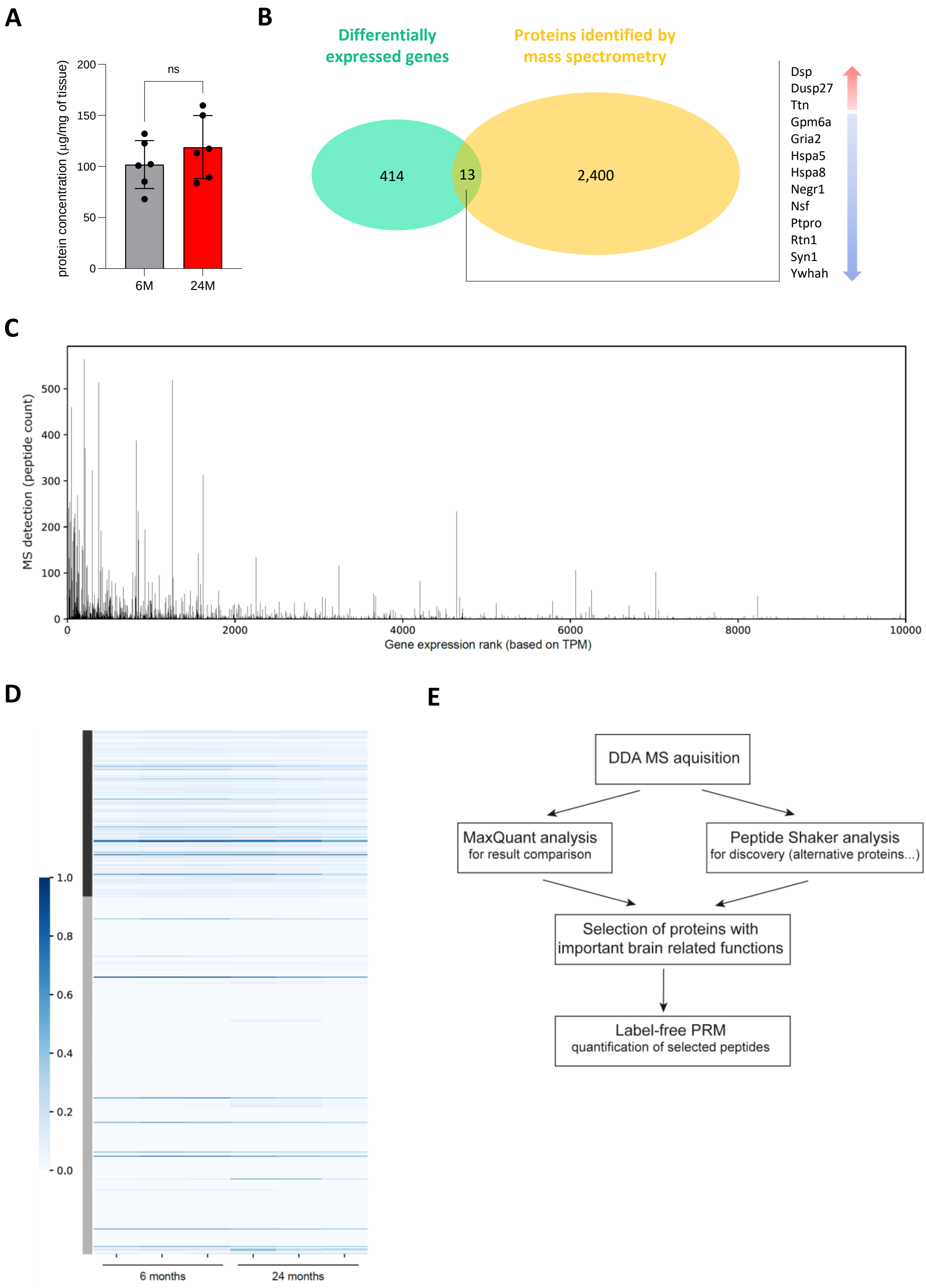

Supplemental Figure S4

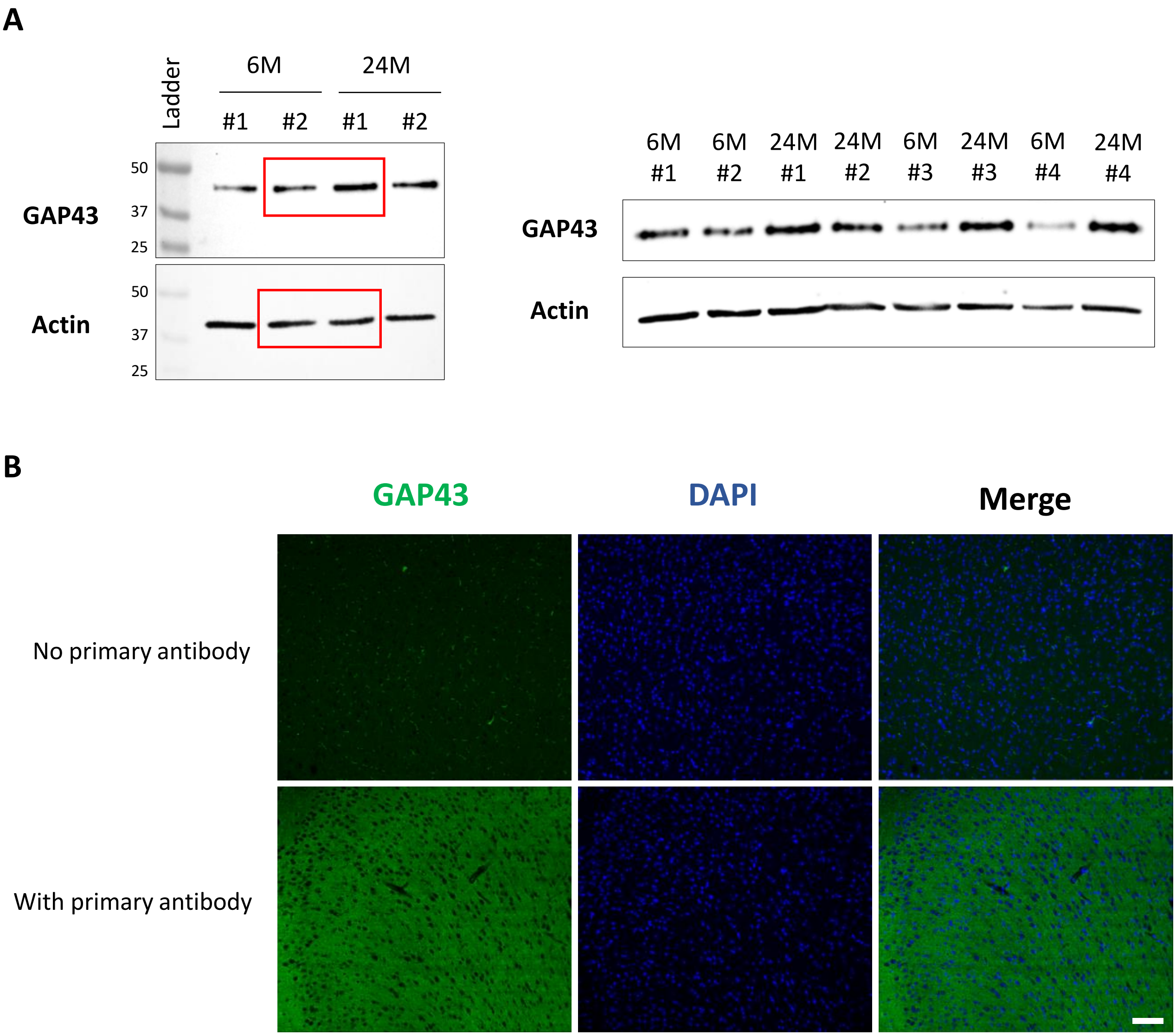

Supplement: Supplementary file 1 [file cells-12-00615-s001.zip › Khatir et al. Supplemental figures.pdf]
