# Supplementary material for: Decoupling of mRNA and Protein Expression in Aging Brains Reveals the Age-Dependent Adaptation of Specific Gene Subsets
Source: Cells. 2023 Feb 14;12(4):615. doi: 10.3390/cells12040615 (PMC9954025; doi:10.3390/cells12040615)
Supplement: Supplementary file 1 [file cells-12-00615-s001.zip › Khatir et al. Supplemental Table S5.pdf]

Supplemental Table S5

DOWN		UP							
Apoa1	Pdcd5	Abi1	Atcay	Dlgap1	Gad1	Lancl2	Pafah1b1	Psmc1	Strap
Apoo	Pin1	Abi2	Atl1	Dmtn	Gad2	Lmnb2	Pak1	Psmc4	Tardbp
Arf5	Ppa2	Acaa2	Bcat1	Dnaja2	Gap43	Lrrc47	Pcbp2	Psmc5	Tceal5
Arl8a	Rpl18	Acad9	Bcs1l	Dnajc11	Gps1	Lsamp	Pcca	Psmd11	Tmx3
C1ql3	Rpl19	Acadl	Bpnt1	Dpysl5	Gsk3a	M6pr	Pccb	Psmd13	Tmx4
Cnksr2	Rpl23a	Acadm	Cacnb3	Dync1li1	Gucy1b3	Maob	Pcyox1l	Psmd3	Tom1l2
Cplx2	Rpl38	Acadvl	Cadm4	Dync1li2	H2afy	Mapk3	Pde1b	Psmd5	Tomm40
Csrp1	Rpl7a	Acot9	Camk2d	Eif3e	Hapln1	Mapre2	Pdia6	Psmd6	Tuba8
Dctn3	Rpl8	Acsl1	Cct5	Eif4a1	Hars	Me3	Pepd	Ptpn9	Txnrd1
Diras1	Rpl9	Actr1b	Cct6a	Eif4a3	Hebp1	Mecp2	Pick1	Rars	Uba3
Dynll2	Rps10	Adk	Cct7	Elavl4	Hexb	Mtch1	Pip4k2a	Rmdn3	Upf1
Enoph1	Rps26	Adrbk1	Cd200	Entpd2	Hnrnpl	Nadk2	Pip4k2c	Rraga	Vat1l
Fabp5	Stmn1	Ahcy	Cd47	Epm2aip1	Hnrnpm	Nap1l4	Pls3	RtcA	Vim
Fam162a	Stmn2	Ak5	Cmpk2	Erlin2	Homer2	Nisch	Pmpca	Ruvbl1	Vps26b
Gng2	Timm9	Aldh6a1	Cndp2	Etf1	Hspa2	Nova1	Ppm1f	Scrn1	Vps45
H2afv	Tmem11	Aldh7a1	Coro2b	Etfdh	Kbtbd11	Nsfl1c	Ppp2r5a	Sh3glb1	Wasf3
Lin7b	Tpm4	Anxa3	Csde1	Fam126b	Kctd12	Nt5dc3	Ppp2r5c	Shroom2	Yars
Lin7c	Ttr	Ap3m2	Cyp46a1	Farsa	Kctd16	Ola1	Ppp5c	Slc9a3r1	
Ndufa7	Txn	Arfip2	Dars	Fdps	Kiaa0513	P4hb	Prkar1a	Smap1	
Nme2	Uchl3	Ass1	Dbnl	Flot2	Klc2	Pa2g4	Prkar2a	Snx5	
Pdap1	Uqcr10	Atad3	Dctn4	Gabra1	Lactb	Pabpc1	Prmt8	Src	
